# Putative circulating adipose tissue-derived stem cells, obesity, and metabolic syndrome features

**DOI:** 10.1007/s40618-023-02067-7

**Published:** 2023-03-23

**Authors:** B. M. Bonora, R. Cappellari, M. Albiero, L. Prevedello, M. Foletto, R. Vettor, A. Avogaro, G. P. Fadini

**Affiliations:** 1 Department of Medicine, University of Padova, 35128 Padua, Italy; 2 Veneto Institute of Molecular Medicine, 35129 Padua, Italy; 3 Bariatric Surgery Unit, University Hospital of Padova, 35128 Padua, Italy

**Keywords:** Weight loss, Bariatric surgery, Remodeling, Mobilization, Migration

## Abstract

**Purpose:**

In mice, adipose tissue-derived stem cells (ASCs) reach the systemic circulation and establish ectopic adipose depots fostering insulin resistance, but whether this occurs in humans is unknown. We examined circulating ASCs in individuals with various combination of metabolic syndrome traits.

**Methods:**

We enrolled patients attending a routine metabolic evaluation or scheduled for bariatric surgery. We quantified ASCs as CD34^+^CD45^−^CD31^−^(CD36^+^) cells in the stromal vascular fraction of subcutaneous and visceral adipose tissue samples and examined the presence and frequency of putative ASCs in peripheral blood.

**Results:**

We included 111 patients (mean age 59 years, 55% males), 40 of whom were scheduled for bariatric surgery. The population of CD34^+^CD45^−^CD31^−^ ASCs was significantly more frequent in visceral than subcutaneous adipose depots (10.4 vs 4.1% of the stromal vascular fraction; p < 0.001), but not correlated with BMI or metabolic syndrome traits. The same phenotype of ASCs was detectable in peripheral blood of 58.6% of patients. Those with detectable circulating ASCs had significantly higher BMI (37.8 vs 33.3 kg/m^2^; p = 0.003) and waist (111.2 vs 105.4 cm; p = 0.001), but no difference in other metabolic syndrome traits (p = 0.84). After bariatric surgery, patients with detectable circulating ASCs had greater BMI reductions at 6 months (− 10.4 vs − 7.8 kg/m^2^; p = 0.014).

**Conclusion:**

Presence of putative circulating ASCs, antigenically similar to those observed in the adipose tissue, is associated with greater adiposity and larger BMI reduction after surgery, but not with clinical signs of metabolic impairment. The role of circulating ASCs in adipose tissue biology and systemic metabolism deserves further investigation.

## Introduction

Obesity leads to metabolic impairment that foster cardiovascular risk [1], but the exact mechanisms linking excess adipose tissue to insulin resistance and metabolic syndrome are incompletely understood [2, 3]. Defective fatty acid trapping and their spill over from dysfunctional adipocytes is thought to spread fat into ectopic depots in metabolically relevant tissues, such as the liver, muscle, and pancreas [4–6]. Lipids can accumulate ectopically within cells of non-adipose origin in the liver and muscle or as a pathologic infiltration of adipocytes in non-adipose tissues such as the pancreas and the muscle or at perivascular sites [7]. Yet, the origin of ectopic adipocytes is controversial [8]. Cells functioning as adipocyte precursors have been identified in several tissues [9], but how they are recruited remains unexplained. In addition, circulating cells may be involved in the development of ectopic fat, though their role is much less appreciated. De novo generation of adipocytes from circulating, bone marrow-derived progenitors has been demonstrated in the murine and human adipose tissue [10]. In humans who received a bone marrow transplant, donor cells contributed to subcutaneous adipocytes, especially in the presence of obesity [11], supporting the existence of a circulating hematopoietic-derived adipocyte progenitor. In mice, adipocyte stem cells (ASCs) from the stromal vascular fraction (SVF) can egress the adipose tissue and migrate through the lymph [12]. In response to a high fat diet, ASCs can leave the subcutaneous adipose tissue and infiltrate skeletal muscle to form an ectopic depot driving metabolic disturbances [13]. Sensing of CXCL12 gradients by CXCR4 signaling has been identified as a regulator of such ASC traffic [13, 14]. Whether this pathway is also active in humans is, at present, completely unknown. We have previously demonstrated an elevation in circulating CD34^+^ hematopoietic stem/progenitor cells (HSPCs) during development of the metabolic syndrome in otherwise healthy middle-aged individuals [15]. Though freshly-isolated tissue ASCs typically express CD34 [16], their contribution to the circulating CD34^+^ cell pool, if any, was considered to be negligible [15]. In addition, ASCs are believed to be of mesenchymal origin and, consequently, should not be engrafted by donor hematopoietic bone marrow transplantation [16, 17].

In this study, we undertook a more thorough evaluation of circulating ASCs in individuals with various combinations of metabolic syndrome traits, some of whom were scheduled for bariatric surgery and had a simultaneous assessment of adipose tissue samples. We aimed to evaluate whether presence of detectable ASCs in the bloodstream associated with obesity and metabolic impairment. We hypothesized that ASCs may appear in the circulation proportionally to the amount of adipose tissue and, if ASCs did contribute to ectopic fat and insulin resistance, such trafficking through the bloodstream would be associated with features of the insulin resistance syndrome.

## Materials and methods

### Patients

The study was conducted according to the principles of the Declaration of Helsinki and approved by the ethical committee of the University Hospital of Padua. Patients were selected among those attending the outpatient clinics of the Department of Medicine (University Hospital of Padova) for evaluation of metabolic diseases or being scheduled for bariatric surgery between October 2015 and October 2017. Male or female patients aged 18–80 years were included if they had at least one metabolic syndrome component, as defined by the ATP-III criteria. Exclusion criteria encompassed any acute clinical condition expected to affect the measure of a circulating stem cell population, such as systemic infection, acute inflammation, cancer, pregnancy and lactation. All patients provided informed consent for blood and tissue sampling, where applicable, and for the use of clinical data for research purposes.

We recorded the following variables: age, sex, height and body weight for the calculation of body mass index (BMI), waist circumference, fasting plasma glucose, HbA1c, lipid profile (total and HDL cholesterol, triglycerides; LDL were calculated using the Friedewald equation), total white blood cell count, history of type 2 diabetes, hypertension, dyslipidemia, and cardiovascular diseases. We also collected information on common medications for the treatment of cardiovascular risk factors.

Outpatients undergoing routine evaluation were subjected to venous blood sampling only. For patients undergoing bariatric surgery, we collected a venous blood sample before the induction of anesthesia and then collected a subcutaneous adipose tissue sample from the abdominal wall and a visceral adipose tissue sample from the omentum. We recorded the change in BMI at routine follow-up visits at 1 and 6 months. Since CXCR4 has been shown to regulate ASC traffic in mice [13], we had the opportunity to analyze circulating ASCs before and after treatment with the CXCR4 antagonist plerixafor in two patients enrolled in the NCT02790957 trial [18].

### Flow cytometry

Omental and subcutaneous adipose tissues were briefly rinsed in ACK buffer to lyse red blood cells (RBC) before removing necrotic tissue and superficial blood vessels. Tissues were minced and placed in 0.1% type I Collagenase (Worthington Biochemical Corporation, NJ, USA) in DMEM/F12 medium (Gibco) for 30 min at 37 °C. Digestion was stopped by diluting collagenase with ice-cold PBS. The digested tissue was dissociated by several passage through a syringe with an 18 G needle until no obvious debris were present. The suspension was passed through a 70 µm cell strainer, centrifuged and residual RBC were lysed with ACK buffer. After centrifugation the pellets were kept on ice until staining.

For preparation of peripheral blood cells, the blood sample was processed within 1 h from collection. 1 mL of blood was lysed with NH_4_Cl, rinsed for 15 min and then centrifuged at 1800 rpm for 5 min.

**Fig. 1:**
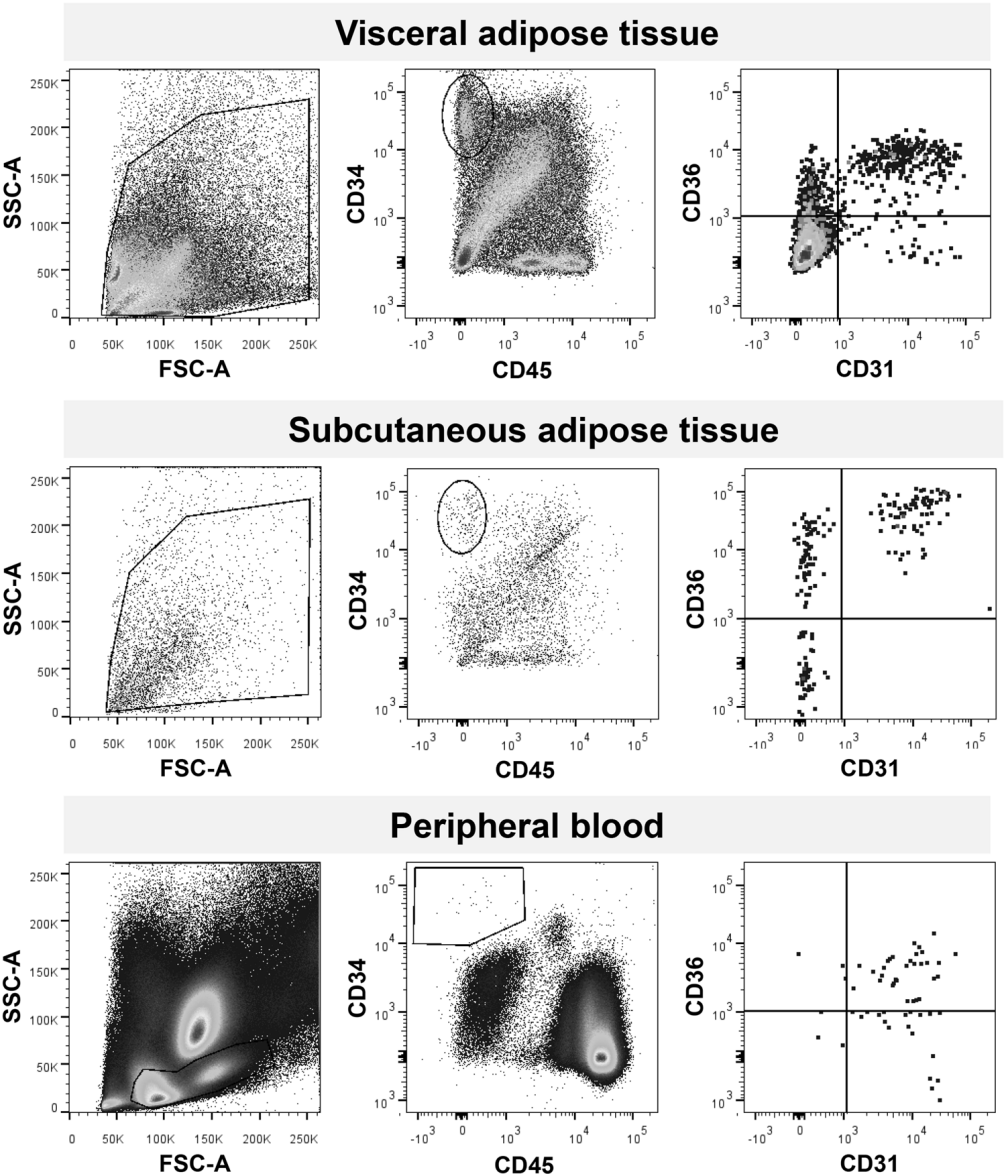
Flow cytometry. Representative FACS plots are shown for the analysis of ASCs in the visceral (upper lane), subcutaneous (middle lane) adipose tissue compartments and in peripheral blood (lower lane). Mononuclear cells were identified in the FSC versus SSC plot (left panels). For adipose tissue, this gate comprised all cells in the stromal vascular fraction). Then, the population of CD34 + CD45- cells were identified (central panels) and examined for the expression of CD36 and CD31 (right panels). ASCs were enumerated as CD34^+^CD45^−^CD31^−^ cells, and the proportion of CD36 expression was quantified

Cells were washed and stained with the following antibodies: 10 μl of mouse anti-human CD45 APC-C7 (BD^®^, clone 2D1, Cat. #348,815); 20 μl of mouse anti-human CD34 PE (BD, clone 8G12, Cat. #345,802); 20 μl of mouse anti-human CD31 FITC (BD Pharmingen, clone WM59, Cat. #555,445); 20 μl of mouse anti-human CD36 GPIIIb (EXBIO, clone TR9, Cat. #1A-451-T100). While setting up the protocol, cells isolated from the SVF were also stained with 7-AAD to verify high viability percentage. At least one million events were acquired in the mononuclear cell (MNC) fraction identified in the side scatter (SSC) versus forward scatter (FSC) plot. We first identified CD45negCD34pos cells, which were then examined for expression of CD31 (ASCs should be negative) and CD36 (which could be positive or negative on ASCs) [16]. In the analysis of the SVF and in some peripheral blood samples, we also stained for CD146 PE (BD Pharmingen, cat #550,315) as an alternative marker to exclude endothelial cells. ASC in the adipose tissue were expressed as % of cells in the SVF, whereas ASC in peripheral blood were expressed as cells / 10^6^ MNCs. The same trained operator performed the analysis during the entire study (Fig. 1).

### Statistical analysis

Continuous variables are expressed as mean and standard deviation, whereas categorical variables are presented as percentage. Normality of continuous variables was checked using the Kolmogorov–Smirnov test and variables significantly deviating from the normal distribution were log transformed before analysis with parametric tests. The comparison between independent groups (e.g. those with versus without detectable circulating ASCs) was performed using two-tail unpaired Student’s t test for continuous variables or chi-square for categorical variables. The comparison of continuous variables between paired samples (e.g. visceral versus subcutaneous adipose tissue from the same individuals) was performed using two-tail paired Student’s t test. Linear correlations were checked using the Pearson’s r coefficient. Change over time in BMI was analyzed using the general linear model for repeated measures. Statistical significance was accepted at p < 0.05 and the Bonferroni correction was applied to account for type I error due to multiple testing. Analyses were ran in SPSS ver. 28 and data were plotted using GraphPad Prism ver 5.

## Results

### Patient characteristics

We included 111 patients, 71 of whom were undergoing routine evaluation for metabolic diseases (mostly diabetes and dyslipidemia) and 40 were undergoing bariatric surgery. Clinical characteristics are presented in Table 1. In this pooled population, average age was 59.0 years, 55% were men, and mean BMI was 35.9 kg/m^2^. Most (70.3%) fulfilled ATP-III diagnostic criteria for the metabolic syndrome with a median (IQR) number of components equal to 3 (2–4). The analysis of circulating ASCs was available for all patients, whereas the analysis of adipose tissue ASC was available only for patients undergoing bariatric surgery. As different findings relate to different subgroups, their clinical characteristics are also presented separately in Table 1. Patients scheduled for bariatric surgery were younger (49.7 vs 64.2 years; p < 0.001), more often women (65.0% vs 33.8%; p = 0.001) and had lower prevalence of diabetes, hypertension, and cardiovascular disease, reflected by less frequent use of drugs for the management of these disorders. The prevalence of metabolic syndrome and the median number of its components were similar in the two groups.

**Table 1:** Clinical characteristics of study participants

Variable	All patients (n = 111)	Routine outpatients (n = 71)	Bariatric surgery (n = 40)	p-value
Age, years	59.0 (11.0)	64.2 (9.6)	49.7 (6.3)	< 0.001
Sex male, %	55.0	66.2	35.0	< 0.001
Menopausal women, (%)	27.9	33.8	17.5	< 0.001
Body mass index, kg/m^2^	35.9 (7.9)	31.5 (5.0)	43.7 (5.9)	< 0.001
Waist circumference, cm	108.8 (9.6)	103.9 (6.5)	117.5 (7.9)	< 0.001
Diabetes, %	63.0	84.5	25.0	< 0.001
Hypertension, %	73.9	83.1	58.0	0.002
Dyslipidemia, %	83.8	83.1	85.0	0.796
Cardiovascular disease, %	39.6	56.4	10.0	< 0.001
Chronic kidney disease, %	11.7	16.9	2.5	0.023
Total cholesterol, mg/dl	182.0 (40.8)	178.6 (42.8)	188.3 (36.5)	0.246
HDL cholesterol, mg/dl	50.2 (15.4)	50.9 (16.0)	48.9 (14.6)	0.511
Triglycerides, mg/dl	139.3 (87.7)	146.2 (94.7)	127.2 (73.3)	0.283
LDL cholesterol, mg/dl	103.5 (35.2)	98.0 (36.3)	113.7 (31.3)	0.028
Fasting glucose, mg/dl	140.9 (43.0)	153.6 (37.9)	118.4 (42.7)	< 0.001
HbA1c, %	7.4 (1.5)	7.5 (1.5)	7.0 (1.9)	< 0.001
White blood cells, 1000/µL	7.1 (2.3)	7.4 (1.6)	6.5 (3.3)	0.08
MS components, median (IQR)	3 (2–4)	3 (2–4)	3 (2–4)	0.961
Aspirin, %	33.3	46.7	10.0	< 0.001
Statin, %	54.1	76.1	15.0	< 0.001
RAS blockers, %	60.4	70.4	43.0	0.003
Insulin, %	24.3	32.3	10.0	0.008
Other glucose lowering drugs, %	55.0	74.6	20.0	< 0.001

Data are presented as mean (standard deviation) or as percentage, where appropriate

*LDL* low density lipoprotein, *HDL* high density lipoprotein, *MS* metabolic syndrome, *IQR* interquartile range, *RAS* renin angiotensin system

### Adipose tissue and circulating ASCs

**Fig. 2:**
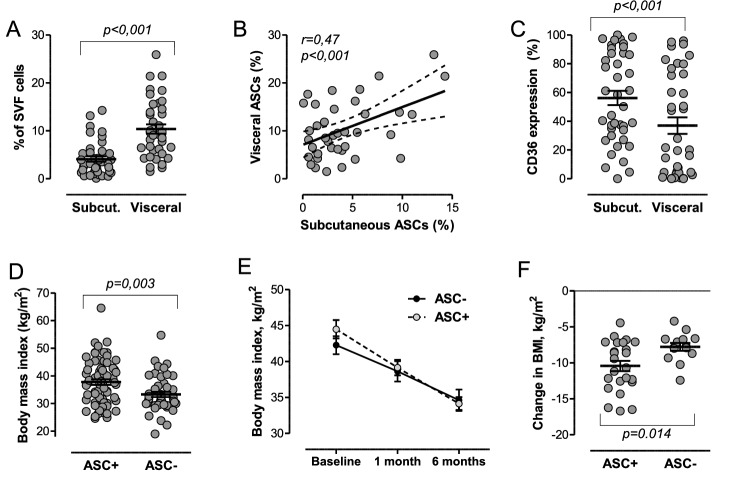
ASC levels and their clinical associations. **A** Comparison between ASCs levels in the subcutaneous versus the visceral adipose tissue. **B** Linear correlation between ASCs percentages in the visceral and subcutaneous adipose tissue of the same individuals. **C** Level of expression of CD36 on ASCs in the subcutaneous versus visceral adipose tissue. **D** Body mass index (BMI) in patients with detectable ASCs in peripheral blood (ASC +) versus those with undetectable ASCs (ASC-). **E** BMI values at baseline, one and six months after surgery in patients with and in patients with detectable ASCs in peripheral blood (ASC +) versus those with undetectable ASCs (ASC-). **F** Total change in BMI at 6 months in the two groups of patients

In the SVF of adipose tissue samples, we identified a distinct population of CD45^−^CD34^+^CD31^−^ cells, which are supposed to represent ASCs (Fig. 1). The population of CD45^−^CD34^+^CD31^−^ ASCs formed an average (± SD) 4.1 ± 3.5% of cells in the subcutaneous adipose tissue SVF and 10.4 ± 6.0% of the visceral adipose tissue SVF. The difference was highly statistically significant in the paired comparison (p < 10^–7^; Fig. 2A). In line with prior studies [19], when reported to the parent CD45^neg^ population of SVF, CD34^+^CD31^−^ cells accounted for ~ 25% and ~ 59% of cells in the subcutaneous and visceral tissue, respectively. There was a significant direct correlation between ASC content in the subcutaneous and visceral adipose tissue (r = 0.47; p < 0.001; Fig. 2B). Percent expression of the membrane fatty acid scavenger CD36 was significantly higher on the surface of ASCs in the subcutaneous that in the visceral adipose tissue (56.2 ± 31.4% versus 37.0 ± 35.5%; p < 0.001 for paired comparison; Fig. 2C). As determined in a subsample of 8 patients, 86% of CD45^−^CD34^+^CD31^−^ ASCs were CD146-negative and the concordance correlation coefficient between CD45^−^CD34^+^CD31^−^ and CD45^−^CD34^+^CD31^−^CD146^−^ cells was 0.997, ruling out any meaningful contamination by endothelial cells.

Among the clinical characteristics listed in Table 1, we found none being significantly associated with ASC content in either the subcutaneous or visceral adipose tissue. We only reported a direct correlation between age and visceral ASC content (r = 0.37; p = 0.011), which was not observed for subcutaneous ASC content. Such correlation was independent from BMI (adjusted r = 0.37; p = 0.02). None of other metabolic syndrome traits significantly correlated with ASCs in either depot, but there was a trend for lower visceral CD45^−^CD34^+^CD31^−^CD36^+^ ASC content in patients with as compared to those without diabetes (1.6 ± 3.6% versus 4.3 ± 4.4%; p = 0.09). Percent expression of CD36 on subcutaneous or visceral ASCs displayed no significant correlation with any clinical characteristic.

Cells with the same phenotype of ASCs quantified in the adipose tissue (CD45^−^CD34^+^CD31^−^) were detectable in peripheral blood of 24/40 patients (60.0%), with a mean ± SD level of 1.9 ± 2.3 cells / 10^6^ MNCs. We found no correlation between ASC levels in peripheral blood and ASC content in either adipose tissue depot, nor there was any difference in adipose tissue ASC content in patients with detectable versus those with undetectable circulating ASCs.

### Clinical features associated with circulating ASCs

The clinical correlates of circulating ASCs were examined in the pooled cohort of patients. Since detection of ASCs was a rare event, we compared patients with detectable ASCs (n = 65; 58.6%) to those with undetectable ASCs in peripheral blood. Therefore, reporting ASCs as absolute count (i.e. cells / ml) in place of a relative count (i.e. cells / million events) is not affecting this patient grouping.

In patients with detectable ASCs, the mean ± SD level was 1.8 ± 1.7 / 10^6^ MNCs and 48.6% expressed CD36. As shown in Table 2, patients with detectable circulating ASCs had significantly higher BMI (37.8 ± 8.3 versus 33.3 ± 6.5 kg/m^2^; p = 0.003) and waist circumference (111.2 ± 9.9 versus 105.4 ± 8.0 cm; p = 0.001) than those with undetectable ASCs, but all other clinical characteristics were similar, including prevalence of other metabolic traits and number of metabolic syndrome components. No association was detected with age and sex. Menopausal women were present only in the bariatric surgery cohort. In such population, no association was detected between detectable circulating ASCs and menopause (p = 0.91).

**Table 2:** Clinical features in relation to the presence or absence of circulating ASCs

Variable	Detectable ASCs (n = 65)	Undetectable ASCs (n = 46)	p-value
Age, years	57.6 (10.2)	60.9 (11.9)	0.111
Sex male, %	58.5	50.0	0.382
Body mass index, kg/m^2^	37.8 (8.3)	33.3 (6.5)	0.003
Waist circumference, cm	111.2 (9.9)	105.4 (8.0)	0.001
Diabetes, %	67.4	52.3	0.388
Hypertension, %	80.4	69.2	0.189
Dyslipidemia, %	81.5	86.9	0.450
Cardiovascular disease, %	47.8	33.8	0.141
Chronic kidney disease, %	10.8	13.0	0.717
Total cholesterol, mg/dl	175.7 (35.8)	190.3 (45.5)	0.068
HDL cholesterol, mg/dl	48.3 (16.3)	52.8 (14.0)	0.141
Triglycerides, mg/dl	137.0 (82.8)	142.4 (94.2)	0.757
LDL cholesterol, mg/dl	99.2 (37.5)	109.2 (38.3)	0.150
Fasting glucose, mg/dl	154.0 (46.3)	141.6 (38.2)	0.060
HbA1c, %	7.5 (1.6)	7.2 (1.5)	0.212
White blood cells, 1000/µL	7.0 (2.3)	7.2 (2.4)	0.623
MS components, median (IQR)	3 (2–4)	3 (2–4)	0.895

Patients were divided in two groups based on whether circulating ASCs were detectable or not in peripheral blood. Data are presented as mean (standard deviation) or as percentage, where appropriate

*LDL* low-density lipoprotein, *HDL* high-density lipoprotein, *MS* metabolic syndrome, *IQR* interquartile range

*Not significant when adjusted for BMI (p = 0.365)

We then checked correlations between ASC levels and clinical features in patients with detectable ASCs. Since the distribution of ASCs was not normal (K-S test = 0.033), the variable was log-transformed. No significant correlation was detected between ASCs and any clinical feature. The percentage expression of CD36 on ASCs was lower in the presence of cardiovascular disease (30.0% versus 59.8%; p = 0.029), with a statistically significance level that would not survive adjustment of type I error inflation due to multiple testing.

Circulating ASC level was analyzed before and 8 h after therapy with the CXCR4 antagonist plerixafor in two patients with diabetes: a man aged 68 with a BMI of 25.3 kg/m^2^ and a woman aged 69 with BMI of 33.6 kg/m^2^. ASCs showed no consistent change: from 1.9/10^6^ MNC before plerixafor to 1.5/10^6^ MNC after plerixafor.

With the flow cytometry data available, it was possible to derive the level of circulating endothelial cells (CECs) as CD45^−^CD34^+^CD31^+^. Consistently with prior literature [20], the levels of CECs were significantly higher in patients with as compared to those without cardiovascular disease (294 ± 206 versus 164 ± 79 cells / mL; p = 0.003).

In the bariatric cohort, after surgery (3 gastric by-pass and 37 sleeve gastrectomy), BMI declined from an average of 43.6 kg/m^2^ to 39.0 kg/m^2^ at 1 month and to 34.3 kg/m^2^ at 6 months (p < 0.001 versus baseline). In repeated-measure analysis adjusted for baseline BMI, patients with detectable ASC showed a significantly greater BMI reduction (p = 0.019; Fig. 1E). Patients with detectable circulating ASCs displayed a greater reduction in BMI than those with undetectable circulating ASCs (− 10.4 kg/m^2^ versus − 7.8 kg/m^2^; p = 0.014; Fig. 1F).

## Discussion

Experimental studies in diet-induced obese mice suggest that ASCs can leave the subcutaneous adipose tissue, seed remote organs and establish ectopic fat depots, thereby contributing to metabolic impairment and insulin resistance. This traffic could be potentially targeted to treat metabolic diseases. We hypothesized that, if this pathway also operated in humans, presence of ASCs in peripheral blood would associate with obesity and the metabolic syndrome, as a consequence of insulin resistance. We show that patients with detectable ASCs in the bloodstream had higher BMI and waist circumference than those with undetectable ASCs, but did not display distinctive signs of metabolic impairment. The CXCR4 axis has been implicated in ASC traffic in mice [13, 14] and it is also the major regulator of HSPCs mobilization in mice and humans [21, 22]. As determined in two patients undergoing HSPC mobilization with the CXCR4 antagonist plerixafor, we found no evidence of ASCs release. This negative finding, though anecdotal, does not confirm the involvement of CXCR4 in a coordinated traffic of ASCs in humans. Therefore, our data so far do not support the concept that ASCs are actively recruited into the circulation by specific pathways and are involved in the development of dysmetabolism. A passive spillover of ASCs from the adipose tissue may be a rare event that explains the association between detectable circulating ASCs and higher BMI and waist. The correlation with waist may indicate that ASCs derive from abdominal (possibly visceral) fat depots, consistent with the finding of higher ASCs frequency in visceral than in subcutaneous adipose tissue. It is also of interest that patients with detectable ASCs in peripheral blood at baseline displayed a greater weight loss than those without circulating ASCs. Therefore, we speculate that leakage of ASC into the circulation may be linked with the capacity of adipose tissue remodeling and shrinkage after surgery.

To analyze circulating ASCs, we used a flow cytometry protocol able to identify ASCs within the adipose tissue SVF. The CD45^−^CD34^+^CD31^−^ phenotype has been suggested as a minimum standard to identify ASCs by the International Federation for Adipose Therapeutics and Science and the International Society for Cellular Therapy [16]. Of note, we found that the proportion of SVF cells with ASC phenotype was more than twofold higher in the visceral than in the subcutaneous adipose tissue. This may reflect the different composition and function of the two adipose depots [23]. Visceral fat was generally thought to have lesser pre-adipocyte differentiation capacity [24], but the mouse visceral adipose was recently found to contain more committed pre-adipocytes and display greater ASC proliferation in response to high fat diet [25]. In addition, functionally different populations of ASCs may be present within the murine and human adipose tissue [25, 26]. A detailed characterization of ASC phenotype was outside the scope of our study, but we found higher expression of the fatty acid transporter CD36 on subcutaneous versus visceral ASCs, which is a proxy of ASCs differentiation capacity [27]. In the visceral adipose tissue, age was directly correlated with the amount of ASCs implying that, at means of BMI, older participants (with a cut point at 50 years of age) had higher ASCs in the visceral adipose tissue. While this might imply a greater adipogenic potential in older individuals, age did not affect CD36 expression on ASCs in either depots and, in the absence of associations with metabolic features, the implications of this finding remain unclear. Patients aged 50 years or older may simply have longer-lasting obesity with greater accumulation of ASCs in the visceral adipose tissue over time. A prior study reported greatly increased ASC content in the subcutaneous and visceral depots of obese versus lean individuals, which was blunted in those with dysglycemia [19]. We found a similar inverse association with diabetes (though not significant), that may imply an impaired adipogenic capacity in the context of dysregulation of glucose metabolism.

Rather than providing a characterization of adipose tissue ASCs, in this study, we aimed to demonstrate that it is feasible to measure ASCs in the blood using the same flow cytometry approach and phenotype that allows ASCs identification within the SVF. The population of CD34^+^ cells in peripheral blood is mostly comprised of CD45^dim^ HSPCs [15], while the majority of CD34^+^CD45^neg^ cells are CECs detached from the vessel wall [28]. ASCs can be distinguished from CECs as being negative for endothelial markers CD31 and/or CD146. Showing high concordance of CD31 and CD146 expression on CD34^+^CD45^−^ cells, we argue that the circulating CD34^+^CD45^−^CD31^−^ MNC phenotype represents putative ASCs. Consistency of this approach and within the clinical database is provided by the observation that levels of CECs were significantly elevated in patients with a history of cardiovascular disease, a notion well established in the literature [20, 29].

At least one ASC out of 10^6^ MNC (~ 0.5 mL of blood) was observed in only 58.6% of patients, and the average number was ~ 2, indicating that presence of circulating ASCs is a rather stochastic phenomenon. Yet, if occurrence of circulating ASCs was a purely random event, there would be no association with clinical characteristics. The associations we observed between presence of circulating ASCs and higher BMI and waist were statistically solid enough to survive the Bonferroni correction of as much as 17 multiple tests (exceeding the number of variables in Table 2) and are therefore unlikely to be chance findings. While recruitment of adipocyte precursors of hematopoietic origin was already found to be related to BMI [10, 11], emergence of ASCs from native depots into the bloodstream is a novel observation. In the cohort of patients undergoing bariatric surgery, association between ASC detectability in peripheral blood and weight loss was also a significant and robust finding.

We acknowledge our study has limitations. First, the subpopulation of patients undergoing bariatric surgery may be small to detect more subtle associations between circulating and adipose tissue ASCs. Second, we do not have definite evidence that antigenically defined cells represent functional ASCs in vitro or in vivo (hence the term “putative”). Furthermore, it is possible that ASCs traffic through the lymph and emerge in the blood only transiently before reaching metabolically active organs. Repeated measures over time in the same individuals may be helpful to clarify this point. In addition, we did not examine ectopic fat depots but only looked at the indirect consequences of insulin resistance that normally results from the ectopic accumulation of lipids. Future studies will need to correlate circulating ASCs with site-specific fat depots. Further details on inflammatory biomarkers beyond the WBC count and on hormonal status may reveal additional insight into the regulation of ASC traffic. Finally, the effect of weight loss after bariatric surgery on circulating ASCs would provide more causality to the association with the degree of adiposity. Notably, an analysis of adipose tissue samples collected before and after bariatric surgery revealed an expansion of ASCs following weight loss [30], which could be seen as an homeostatic response to shrinkage of the adipose tissue. Whether circulating ASCs mirror such response is of interest and should be investigated in future studies.

In conclusion, patients with circulating ASCs have higher BMI and waist circumference than those with undetectable ASCs in peripheral blood but display no clear evidence of metabolic dysregulation. When subjected to bariatric surgery, patients with detectable ASCs in peripheral blood lost more weight. These findings suggest that greater adiposity may favor the release of ASCs into the bloodstream and, possibly, enhance adipose remodeling and shrinkage after surgery. Data so far do not support an active role for ASC traffic in the development of the metabolic syndrome, but further studies in humans are needed to explore this fascinating cellular pathway.

## Data Availability

Restrictions apply to the availability of original data used in this study, owing to privacy issues and local policies. The corresponding author will on request detail the restrictions and any conditions under which access to some data may be provided.
